# Horizontal acquisition of hydrogen conversion ability and other habitat adaptations in the Hydrogenovibrio strains SP-41 and XCL-2

**DOI:** 10.1186/s12864-019-5710-5

**Published:** 2019-05-06

**Authors:** Giorgio Gonnella, Nicole Adam, Mirjam Perner

**Affiliations:** 1 Universität Hamburg, MIN-Fakultät, ZBH - Center for Bioinformatics, Bundesstraße 43, Hamburg, 20146 Germany; 2 GEOMAR Helmholtz Center for Ocean Research Kiel, Geomicrobiology, Wischhofstr. 1-3, Kiel, 24148 Germany; 3 previous address: Universität Hamburg, MIN-Fakultät, Biocenter Klein Flottbek, Molecular Biology of Microbial Consortia, Ohnhorststr. 18, Hamburg, 22609 Germany

**Keywords:** Hydrogenase, Hydrogenovibrio, Horizontal gene transfer, Hydrothermal vents, Habitat adaptation, Bacterial genome, Genome comparison

## Abstract

**Background:**

Obligate sulfur oxidizing chemolithoauthotrophic strains of *Hydrogenovibrio crunogenus* have been isolated from multiple hydrothermal vent associated habitats. However, a hydrogenase gene cluster (encoding the hydrogen converting enzyme and its maturation/assembly machinery) detected on the first sequenced *H. crunogenus* strain (XCL-2) suggested that hydrogen conversion may also play a role in this organism. Yet, numerous experiments have underlined XCL-2’s inability to consume hydrogen under the tested conditions. A recent study showed that the closely related strain SP-41 contains a homolog of the XCL-2 hydrogenase (a group 1b [NiFe]-hydrogenase), but that it can indeed use hydrogen. Hence, the question remained unresolved, why SP-41 is capable of using hydrogen, while XCL-2 is not.

**Results:**

Here, we present the genome sequence of the SP-41 strain and compare it to that of the XCL-2 strain. We show that the chromosome of SP-41 codes for a further hydrogenase gene cluster, including two additional hydrogenases: the first appears to be a group 1d periplasmic membrane-anchored hydrogenase, and the second a group 2b sensory hydrogenase. The region where these genes are located was likely acquired horizontally and exhibits similarity to other *Hydrogenovibrio* species (*H. thermophilus* MA2-6 and *H. marinus* MH-110 ^*T*^) and other hydrogen oxidizing Proteobacteria (*Cupriavidus necator* H16 and *Ghiorsea bivora* TAG-1 ^*T*^). The genomes of XCL-2 and SP-41 show a strong conservation in gene order. However, several short genomic regions are not contained in the genome of the other strain. These exclusive regions are often associated with signs of DNA mobility, such as genes coding for transposases. They code for transport systems and/or extend the metabolic potential of the strains.

**Conclusions:**

Our results suggest that horizontal gene transfer plays an important role in shaping the genomes of these strains, as a likely mechanism for habitat adaptation, including, but not limited to the transfer of the hydrogen conversion ability.

**Electronic supplementary material:**

The online version of this article (10.1186/s12864-019-5710-5) contains supplementary material, which is available to authorized users.

## Background

*Hydrogenovibrio crunogenus* (recently reclassified by [1]) was originally isolated from a deep-sea hydrothermal vent and described as the sulfur-oxidizing *Thiomicrospira crunogena*, belonging to the Gammaproteobacteria [2]. Since then numerous strains, tentatively assigned by phylogenetic analyses to this species, have been isolated from ubiquitous deep-sea hydrothermal vents: e.g. TH-55 ^*T*^ [2] from the Eastern Pacific Rise; L-12 [3] and XCL-2 [4] from the Galapagos Rift, Eastern Pacific; HY-62 [5] from the North Fiji Basin, Western Pacific; 37-SI-2 [6] from the Yonaguni Knoll IV field in the Western Pacific; MA-3 [7] from the Trans-Altantic Geotraverse. A close relative of *H. crunogenus* XCL-2, according to a 16S rRNA gene-based phylogenetic analysis [8], is strain SP-41, isolated from low-temperature fluids, collected near the Sisters Peak chimney, Mid-Atlantic Ridge [9].

For many years, members of the *Thiomicrospira* lineage, now reclassified into *Hydrogenovibrio*, *Thiomicrospira* and *Thiomicrorhabdus* [1], were considered to be indicators for sulfur cycling, as they were known to oxidize reduced sulfur compounds, such as hydrogen sulfide, thiosulfate and tetrathionate ([10], e.g.). The only species among this clade known to oxidize hydrogen was *Hydrogenovibrio marinus* [11, 12], while other phylogenetically closely related organisms remained recalcitrant for many years towards cultivation with hydrogen ([13], and references therein). It was not until the genome of XCL-2 was sequenced, that first indications for alternative energy sources were suggested, based on the hydrogenase genes found on the chromosome [14]. More recently the ability throughout this group to oxidize hydrogen was demonstrated for several strains [8, 15] explaining why some of these organisms are found in relatively sulfide low but hydrogen rich vent systems, such as Lost City, where metagenomic sequences highly similar to the XCL-2 genome were observed [16]. Intriguingly, although the genome of XCL-2 includes a complete set of genes for the assembly and maturation of a [NiFe]-hydrogenase (EC 1.12.1.2) [14], the strain is unable to grow on hydrogen, under all tested conditions [8]. For some of the *H. crunogenus* strains (SP-41, TH-55 ^*T*^) capable of oxidizing hydrogen, it has been shown that this ability depends on the concentrations of Ni and Fe in the medium (strains SP-41 and TH-55 ^*T*^ [13]), but in case of XCL-2 this supplementation made no difference with respect to its hydrogen consumption ability [8].

Hansen and Perner [8] cloned and characterized the [NiFe]-hydrogenase large subunits from several *Hydrogenovibrio* and *Thiomicrospira* strains (MA2-3, L-12, JB-B2, TH-55 ^*T*^) and compared the gene order of the hydrogenase gene cluster in XCL-2 to that of related organisms. Their analysis lead to several hypotheses about why XCL-2 cannot but other phylogenetically closely related strains can indeed utilize hydrogen. Important features missing from XCL-2 could be a membrane-anchoring cytochrome b subunit, a Tat-signal and proper [Fe-S]-cluster binding sites in the small subunit. However, as, until now, no genome sequence has been available for hydrogen consuming strains assigned or closely related to *H. crunogenus*, it has not been possible to verify these hypotheses. Nevertheless, the genomes of several other members of the genera *Hydrogenovibrio*, *Thiomicrospira* and *Thiomicrorhabdus* have been sequenced [17–21]. A recent comparative genomic analysis focused on 18 strains of these genera [21] and identified several genomic features responsible for adaptations to habitat variability of these organisms, likely explaining their cosmopolitan distribution. Horizontal acquisition was suggested for the hydrogenase genes present in some strains of *H. thermophilus* and *H. marinus*, due to their uneven distribution throughout the members of these genera, and the presence of phage genes in juxtaposition to the hydrogenase gene cluster. However, the differences throughout *H. crunogenus* strains remained puzzling, as a hydrogenase gene cluster is contained also on the XCL-2 genome, and the study does not include other strains of *H. crunogenus*.

We present here the complete genome sequence of the hydrogen consuming strain SP-41, and compare it to the genome of XCL-2. Our analysis shows that additional, likely horizontally acquired, genetic material in each of the strains, reflect probable adaptations of these organisms to their habitats. This includes an additional hydrogenase gene cluster in SP-41, which could explain the different hydrogen consumption abilities of the two strains.

## Results and Discussion

### Sequencing and annotation of the SP-41 genome

The genome assembly of the isolated SP-41 was obtained using the Pacific Biosciences platform. The reads (total length of 1.24 Gbp, 503X coverage) were assembled into a single 2.47 Mbp long contig. Illumina sequences previously obtained from the enrichment culture from which SP-41 was isolated were mapped to the assembly and used for 192 corrections (mostly of single nucleotides).

**Fig. 1:**
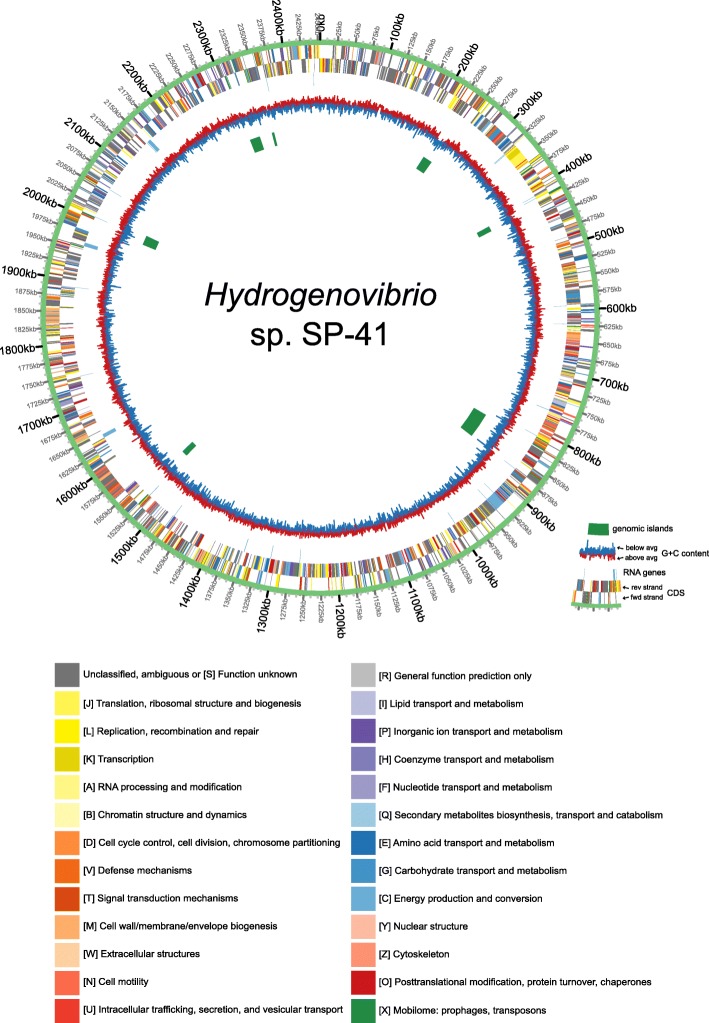
Circos plot of the genome of *Hydrogenovibrio* sp. SP-41. From the outside to theinside, the plot contains the following tracks: CDS (plus strand); CDS (minus strand); RNA genes (both strands); G+C content; genomic islands. The CDS are colored according to the COG annotation (functional categories, see legend on the right)

The final assembly is 2’453’259 bp long. The genome includes 9 rRNA genes (3 copies of 23S, 16S and 5S), 44 tRNA genes (for all canonical amino acids), a tmRNA gene and 2293 protein coding genes (Fig. 1). 87.3% of the protein products had a specific annotation (i.e. not “hypothetical protein”) and 37.5% were assigned an EC number by Prokka [22]. COG annotations by CD-search [23] were assigned to 64.0% of the proteins (Additional file 1), and KO annotations by Blastkoala [24] to 64.2% of the proteins (Additional file 2).

### Phylogenetic relation to other strains

Analysis of the average nucleotide identity (ANI), shows that the strain most closely related to SP-41, for which a genome sequence is available, is XCL-2 (Additional file 3). Most proteins encoded by the SP-41 genome (2019 or 88.1%) have a homolog in XCL-2, in most cases (1986) encoded by a gene in the same position of the alignment of the two genomes (Additional file 4). In both strains, a similar proportion of proteins have no ortholog in the other strain (11.9% of proteins of SP-41; 11.4% XCL-2). In SP-41, these exclusive proteins are more often uncharacterized: 28.8% of them are hypothetical proteins in SP-41, 10.6% in XCL-2.

The relatedness of SP-41 and XCL-2 was already suggested by a previous partial sequencing of the 16S rRNA gene of SP-41 (Genbank KJ573628), in which only 3 differences were found compared to the 16S rRNA gene of XCL-2 (which itself has 3 identical 16S copies) [9]. The 16S rRNA gene sequences obtained from the genome sequencing of SP-41 confirm one of the 3 differences, located in the V7 hypervariable region, present in all three 16S rRNA gene copies of SP-41 (Additional file 5). The further two differences previously observed are not confirmed by the genome sequencing (they are located in the forward sequencing primer region and were likely a sequencing artefact). However, the genome sequence of SP-41 also highlighted the intragenomic heterogeneity of its 16S rRNA genes. Through the genome sequencing, it became clear that the previously published 16S rRNA gene sequence of SP-41 actually represented a consensus sequence, as each of the copies have additional differences, compared to XCL-2, not present in the other two copies (and not present in the previously published sequence). The differences in single copies were masked in the sequencing by the other two copies and lead to wrong base callings (Additional file 6). These are located in the V1 region (1 difference, 1st 16S rRNA gene copy) and V2 region (4 differences, 2nd 16S rRNA copy; 2 differences, 3rd 16S rRNA gene copy). The high level of 16S rRNA gene identity between SP-41 and XCL-2 is thus confirmed, ranging from 99.7% to 99.9% depending on which SP-41 16S rRNA gene copy is considered.

Phylogeny reconstruction based on the 16S rRNA genes [8] assigned several strains to the *H. crunogenus* species: TH-55 ^*T*^, SP-41, XCL-2, L-12, EPR75, 37-SI-2, MA-3, HY-62. However, the comparison of the genome sequences of SP-41 and XCL-2 shows that these two strains belong to two different species, as their ANI of 87.7% is significantly below the 95% threshold suggested for species definition [25]. Despite this, the two strains are the closest relative of each other for which a genome sequence is available (Additional file 3). For the other strains mentioned before, only the sequences of the 16S rRNA gene and in some cases of the *hynL* gene are available [8]. Without further genome sequences it is thus not possible to accurately reconstruct the phylogeny of the lineage and, in particular, to understand, if the other strains previously assigned to *H. crunogenus* should be assigned to the same species as SP-41.

### Genomic structure and plasticity

**Fig. 2:**
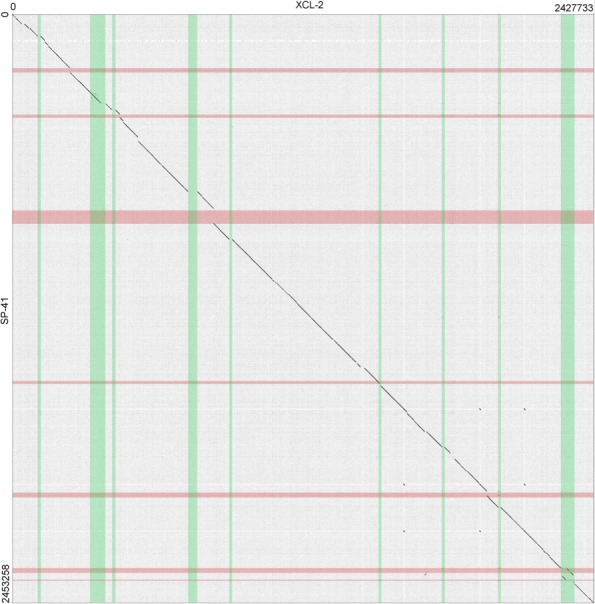
Dotplot representation of the alignment of the genomes of *Hydrogenovibrio crunogenus* XCL-2 and *Hydrogenovibrio* sp. SP-41. The regions of the genome predicted to be genomic islands are highlighted in green (for XCL-2) and red (for SP-41). Counting from the lowest coordinate, islands 1, 3, 4, 5, 7, 8 of XCL-2 and islands 1, 2, 3, 5 of SP-41 contain mostly exclusive sequences, thus were likely acquired after the divergence of the two strains. An island is in common (Island 6 of XCL-2 / 4 of SP-825 41). Island 2 of XCL-2 is partially common to SP-41, but not predicted as an island in that strain. Island 9 of XCL-2 partially overlaps islands 6 and 7 of SP-41, in a region of lower similarity, compared to the rest of the alignment (a translocation is also located there)

The genome of SP-41 is slightly larger (25.5 kbp or 1.0% more) than that of XCL-2. The dot plot of their alignment of the genome sequence of SP-41 to that of XCL-2 (Fig. 2) shows that most regions of the two genomes are homologous and collinear (in total 2.17 Mbp, 88.6% of the SP-41 genome; 89.7% XCL-2). The remaining parts of the alignment (Additional file 7) include (a) 61 exclusive regions, where one genome contains a sequence with at least one annotated feature (36 regions in SP-41 and 25 in XCL-2), and the other genome contains either no sequence or a non-homologous sequence with no features; (b) 12 divergent regions, non-homologous and containing at least one feature in both genomes; (c) a single translocation, i.e. homologous region of 12.1 kbp which is located 50 kbp ahead in the SP-41 sequence, with respect to the rest of the aligned sequences.

The alignment does not reveal if the additional sequences found in exclusive and divergent regions represent sequences lost in the other strain, compared to the last common ancestor, or acquired by horizontal transfer. Thus, we analyzed the two genomes using IslandViewer [26] to identify genomic islands. In total, 7 islands were found in SP-41 (Additional file 8), between 4.5 kbp and 51.6 kbp in length, coding for a total of 148 protein-coding genes (Additional file 9). For comparison, XCL-2 contains 9 islands, between 7.6 kbp and 64.8 kbp, with a total of 377 protein-coding genes (Additional file 10).

In the dot plot in Fig. 2, we highlighted the coordinate ranges of the islands in SP-41 (green) and XCL-2 (red), to allow a visual identification of the overlap of islands of the two genomes and of the islands with exclusive regions. The results show that only part of the islands overlap with exclusive regions of the two genomes: 13 exclusive regions in SP-41 and 12 regions in XCL-2. This appears to be more consistent to a loss of sequences after strain divergence for the remaining exclusive regions.

All genomic island prediction software is based on heuristics, which might fail to find the exact island boundaries or even miss entire islands in some cases. The island annotation by IslandViewer is based on the combination of two programs: IslandPath-DIMOB which looks for dinucleotide biases in a region of at least 8 consecutive genes, including a mobility gene [27]; Islander which looks for regions flanked by a tRNA gene or a tRNA gene fragment and containing an integrase gene. The limitations in predicting genomic islands are shown by the second island of XCL-2, which is largely homologous to SP-41, where, however, no island is predicted in the region. Furthermore, proteins related to transposition are present in other 5 of SP-41 exclusive regions not overlapping predicted islands (while in XCL-2 they are present only in predicted genomic islands), indicating possible further islands not recognized by the prediction software.

As discussed in [14], XCL-2 contains a prophage sequence, which is not present in SP-41, and represents the largest exclusive region of XCL-2 (38.7 kbp) in the alignment to SP-41. However, in general, SP-41 shows a trend towards more genome plasticity: It contains more exclusive regions (36 vs 25), although of slightly smaller average size (5.6 vs 6.4 kbp) than the exclusive regions of XCL-2. Furthermore, 30 SP-41 but only 3 XCL-2 proteins were annotated with transposase/putative transposase KO (K07483;K07497) and/or COGs (COG2801;COG2963;COG3328). In environments associated with hydrothermal venting, a high prevalence of transposases has been previously observed in the biofilm coating the carbonate chimneys of Lost City [28]. There it has been hypothesized to serve as a generator of phenotypic diversity as counterpart to the low organismal diversity of the biofilm community, and possibly contributing to its overall fitness. Both strains XCL-2 and SP-41 were isolated from hydrothermally influenced samples. Thus it remains unclear, which other factors explain the presence and abundance of transposases in one, but not in the other strain.

A 6.0 kbp exclusive region of SP-41 contains a CRISPR array, with 22 repeat units, and the associated proteins Cas1, Cas2 and Cas9. CRISPRs are thought to confer immunity towards invading DNA, such as plasmids and viruses, matching the spacers’ sequences [29]. This could be more useful in habitats where DNA mobility is more common. To understand if the presence of a CRISPR could be correlated to the abundance of transposases observed in SP-41, we counted the number of transposases and CRISPRs in a group of autotrophic Proteobacteria genomes previously analysed by [21]. We found that, in these organisms, the average number of transposases is significantly higher (p-value 0.02) in genomes with annotated CRISPRs (27.3 transposases in average) than in those where no CRISPR is present (14.6 transposases in average) (Additional file 11). However, e.g. within the *Thiomicrospira*/*Hydrogenovibrio*/*Thiomicrorhabdus* lineage this is not always the case. *Hydrogenovibrio* sp. Milos-T1 and *Thiomicrorhabdus* sp. Milos-T2 have a high number of transposases [21], but a CRISPR was annotated only in Milos-T1. Other members of the lineage containing a CRISPR (*Hydrogenovibrio halophilus* DSM 15072 ^*T*^, *Hydrogenovibrio marinus* MH-110 ^*T*^, *Hydrogenovibrio* sp. MA2-6, *Thiomicrorhabdus* sp. Kp2, *Thiomicrospira aerophila* AL3 ^*T*^, *Thiomicrospira microaerophila* ASL8-2 ^*T*^) do not generally show a high number of transposases.

### Hydrogenase gene clusters

The hydrogenase gene cluster (encoding the structural hydrogenases, catalyzing *H*_2_⇔2*H*^+^+2*e*^−^ as well as accessory, assembly and maturation proteins) of XCL-2 is also found in SP-41 (genes GHNINEIG_02156 to GHNINEIG_02165). For ease of reading we name it hydrogenase gene cluster I. The hydrogenase belongs to group 1b [30]. The gene for the large subunit had been previously cloned and characterized [8]. The small subunit has the same unusually large size, as in XCL-2 (813 aa). The entire cluster is present also in SP-41, with the same gene order as in XCL-2.

Besides the XCL-2 resembling hydrogenase gene cluster, a further hydrogenase gene cluster is located on the SP-41 genome (here named hydrogenase gene cluster II). It is part of the largest exclusive region of the SP-41 genome (relative to XCL-2) with 62.6 kbp (starting at position 808620). In total, this exclusive region contains 63 protein-coding genes. Up- and downstream of this region are genes involved in DNA mobilization and modification. A horizontal acquisition of this region is supported by the genomic island prediction, which covers a large part of the area (the last 50.4 kbp). The hydrogenase gene cluster and some related genes (described below) are contained in the central part of the region (27 genes, from gene GHNINEIG_00794 to gene GHNINEIG_00820).

The first of the two hydrogenases from the hydrogenase gene cluster II is encoded by genes GHNINEIG_00797 (large subunit) and GHNINEIG_00798 (small subunit). The small subunit contains the Tat motif RRXFXK important for the translocation to the periplasm [31]. This motif is absent in the other two small subunits from the hydrogenase gene cluster I encoded on both SP-41 and XCL-2 genomes. Furthermore, the presence of a cytochrome b subunit gene (gene GHNINEIG_00796) on the hydrogenase gene cluster II suggests anchoring of the hydrogenase to the membrane [32]. In contrast, this gene is not present in hydrogenase gene cluster I and in its homolog in XCL-2. SP-41 hydrogenase activity was shown to be localized in the membrane and not in the soluble fraction [9]. The lack of the Tat motif and Cytochrome b subunit was postulated to be a possible reason for the hydrogenase inactivity, under the tested conditions, of the XCL-2 hydrogenase [8]. Their presence here could explain why SP-41 is able to consume hydrogen, while XCL-2 is not.

Sequence motifs of the hydrogenases encoded by genes GHNINEIG_00797 and GHNINEIG_00798 resemble hydrogenases assigned to group 1d [30]. In particular, the large subunit (gene GHNINEIG_00797) contains L1 (VERICGVCTGCH) and L2 (SFDPCLACSTH) motifs compatible with the group 1d classification [30]). Interestingly, the L3 (HDHIVHFYHLHALD) and L4 motifs (GTVAAPRGALAH) are the canonical motifs, i.e. those not found in the XCL-2 hydrogenase large subunit and its ortholog in SP-41. The small subunit (gene GHNINEIG_00798) contains proximal and distal cluster binding motifs typical of group 1d, while the 5th position of the medial binding motif (FPIQAGHGCIGCS) contains an Alanine instead of a Serine of the described motif for group 1d (xPIxSGHxCxGCx) and is compatible to group 1f.

The second hydrogenase in the hydrogenase gene cluster II is encoded by genes GHNINEIG_00818 (large subunit) and GHNINEIG_00819 (small subunit). Its small subunit does not contain a Tat-motif. Its medial and distal cluster binding motifs are compatible with group 2b, while the proximal cluster binding motif contains a Serine instead of Glycine at its third position, when compared to the motif described for group 2b (xCGGCx—xCxxxGG—xCP). The large subunit contains L1 (APRICGICSVSQ) and L2 (SFDPCMVCTVH) motifs compatible with this group assignment. This suggests a sensory function for this hydrogenase [30]. The following gene (GHNINEIG_00820) is an homolog of the *Escherichia coli* K12 *zraS*/*hydH* gene. This is a sensory protein kinase [33], originally described as regulating the labile hydrogenase activity in *E. coli* K12 [34] and is also homologous to the HoxJ component of the hydrogen-sensing system of *Cupriavidus necator* (formerly *Alcaligenes eutrophus*) [35].

### Function of the hydrogenase clusters

**Fig. 3:**
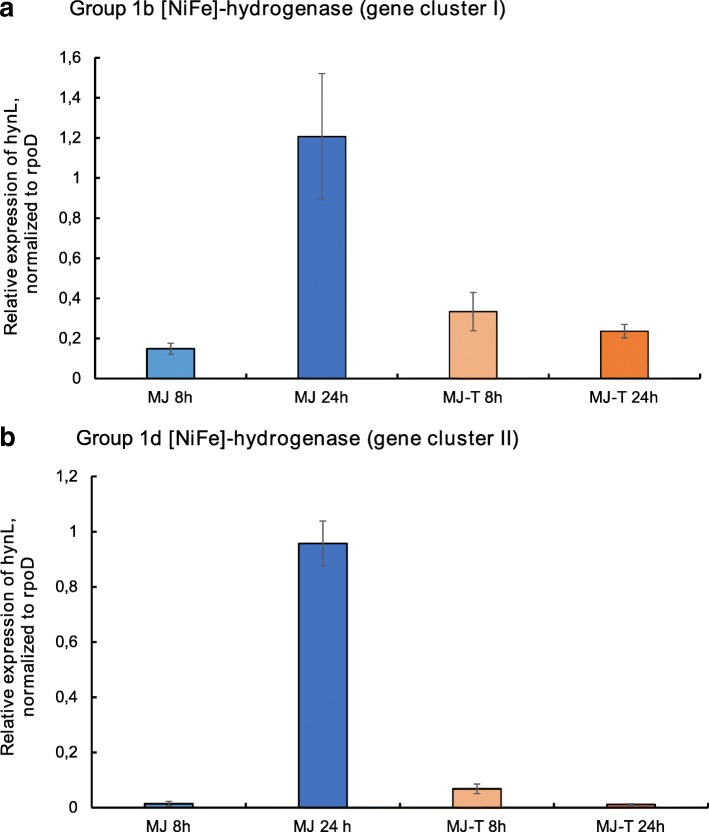
Expression of SP-41’s hydrogenase genes under different cultivation conditions. Relative expression of the *hynL* genes for the large subunit group 1 [NiFe]-hydrogenases in hydrogenase gene cluster I **a** and cluster II **b**, normalized to the housekeeping gene *rpoD*. The expression was analyzed for SP-41 grown in MJ medium for 8 h (light blue bars) and 24 h (dark blue bars), in MJ-T medium grown for 8 h (light orange bars) and 24 h (dark orange bars)

In order to test the expression of the two group 1 [NiFe]-hydrogenases (group 1b, encoded by hydrogenase cluster I, and group 1d, encoded by hydrogenase cluster II), we performed qRT-PCR experiments with RNA extracts of SP-41, grown with an atmosphere of H_2_:CO_2_:O_2_:He(2:20:1:78*%*(*v*/*v*)). Both cluster I and cluster II [NiFe]-hydrogenases are expressed in SP-41 under the tested cultivation conditions, i.e. with (MJ-T medium) and without (MJ medium) thiosulfate addition (Fig. 3). For both hydrogenases the highest expression levels are observed after 24 h and if hydrogen is the only available electron donor. If thiosulfate is available in the medium, the relative expression of both hydrogenase genes is significantly lower. In contrast to the MJ incubation, in the thiosulfate supplemented MJ-T medium the highest expression levels of both hydrogenases is observed after 8 h incubations. For the cluster I hydrogenase, this was already shown in [9]. This effect is most obvious for the cluster II hydrogenase, which altogether exhibits considerably lower expression levels than the cluster I hydrogenase in the MJ-T incubations.

It was previously observed that membrane fractions of hydrogen-oxidizing *Hydrogenovibrio* strains containing a group 1b hydrogenase (e.g. SP-41) display a higher hydrogenase activity rate than those containing a group 1d hydrogenase (MA2-6, MH-110) [8]. However, the presence of the group 1b hydrogenase alone (in XCL-2) does not confer the ability to oxidize hydrogen under all tested conditions. Also the presence of a group 1d hydrogenase alone does not necessarily explain all aspects of the observed hydrogenase activity. E.g. although both strains contain a group 1d hydrogenase, the hydrogen affinity of the hydrogenases of MA2-6 and SP-41 appears to be different: MA2-6 consumes initially more H_2_, but its activity ends at higher H_2_ concentrations [8].

The degree of sequence conservation, and gene expression in SP-41, suggest that also cluster I genes do actually encode a structural hydrogenase [8]. As both hydrogenases are expressed simultaneously during the observed hydrogen consumption activity, it is possible that some components of hydrogenase gene cluster II, missing or not functional in cluster I, affect the other cluster. As no hydrogenase activity was detected in soluble fractions [8], this would require anchoring of the hydrogenase of cluster I to the membrane by components of cluster II, e.g. by its cytochrome b subunit. Some chaperons and maturation proteins of cluster II might as well affect the cluster I hydrogenase. Also, the group 2b sensory hydrogenase might regulate the expression of the cluster I hydrogenase. A similar regulation mechanism appears to affect both hydrogenases, as the expression levels of both hydrogenases correlate well (Fig. 3). Further experiments will be necessary to test these hypotheses.

Despite a possible interaction, the cluster I hydrogenase is likely to function also independently from cluster II as a soluble hydrogenase and/or with a different regulation mechanism, under other currently undetermined environmental conditions. Cluster I is located far on the genome from cluster II, and is absent in XCL-2, where it is still well conserved. Other strains exhibiting high hydrogenase oxidation activity in their membrane fraction, such as TH-55 ^*T*^, MA-3 and L-12 have a group 1b hydrogenase. However, their genomes have not yet been sequenced, thus it is unknown if these strains also contain further hydrogenases, as SP-41.

Both XCL-2 and SP-41 are microaerophiles, suggesting that they are able to use O_2_ as electron acceptor. Similar to the other members of the lineage [21], including XCL-2, SP-41 carries the genes for a cbb_3_-type cytochrome oxidase (EC 1.9.3.1). This enzyme is found mostly in Proteobacteria, but with representants spread across all bacterial phyla [36], and is typically expressed under microaerophilic conditions [37]. Besides oxygen, hydrogen oxidation can be coupled to the reduction of several other molecules [38]. Therefore, we tried to identify genes, which could suggest the potential use of alternative electron acceptors. A nitrate reductase gene is annotated in SP-41 and XCL-2, but it is not likely to have a respiratory function: denitrification tests on *H. crunogenus* TH-55 ^*T*^ were negative [2]. Five genes are homologs of *dsrE*, a component of dissimilatory sulfite reductase systems. However, DsrE may as well be involved also in sulfate oxidation [39], and no further component of a Dsr system is found in the genome. Thus, similar to what was previously noted for XCL-2 [14], we conclude that no other known terminal oxidase, besides cbb_3_ is present in SP-41.

### Homologs of the hydrogenase gene cluster II proteins

**Fig. 4:**
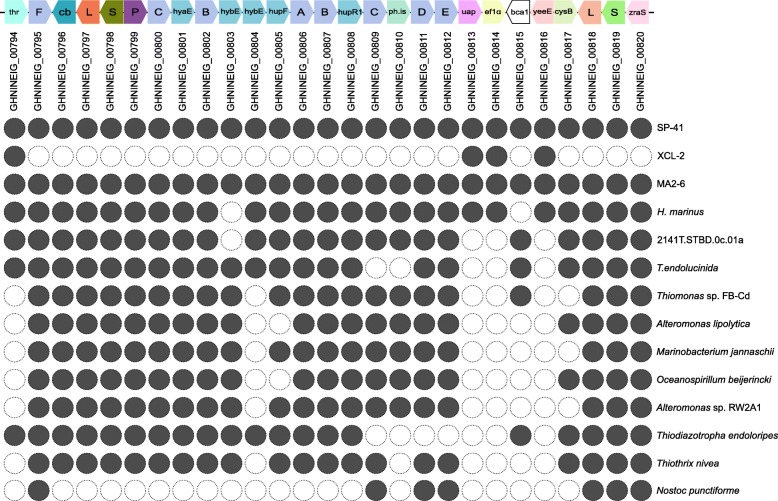
Presence of homologs in other organisms of the proteins encoded by Hydrogenase gene cluster II of SP-41. Diagram showing for which genes in the SP-41 hydrogenase gene cluster not present in XCL-2 (genes GHNINEIG_794 to GHNINEIG_820) homologs were found (full circle) or not (empty circle) in several organisms using BlastP. The results for XCL-2, bacteria with 20 or more homologs and the bacterium outside of Proteobacteria with the highest number of homologs (*Nostoc punctiforme*) are shown

**Fig. 5:**
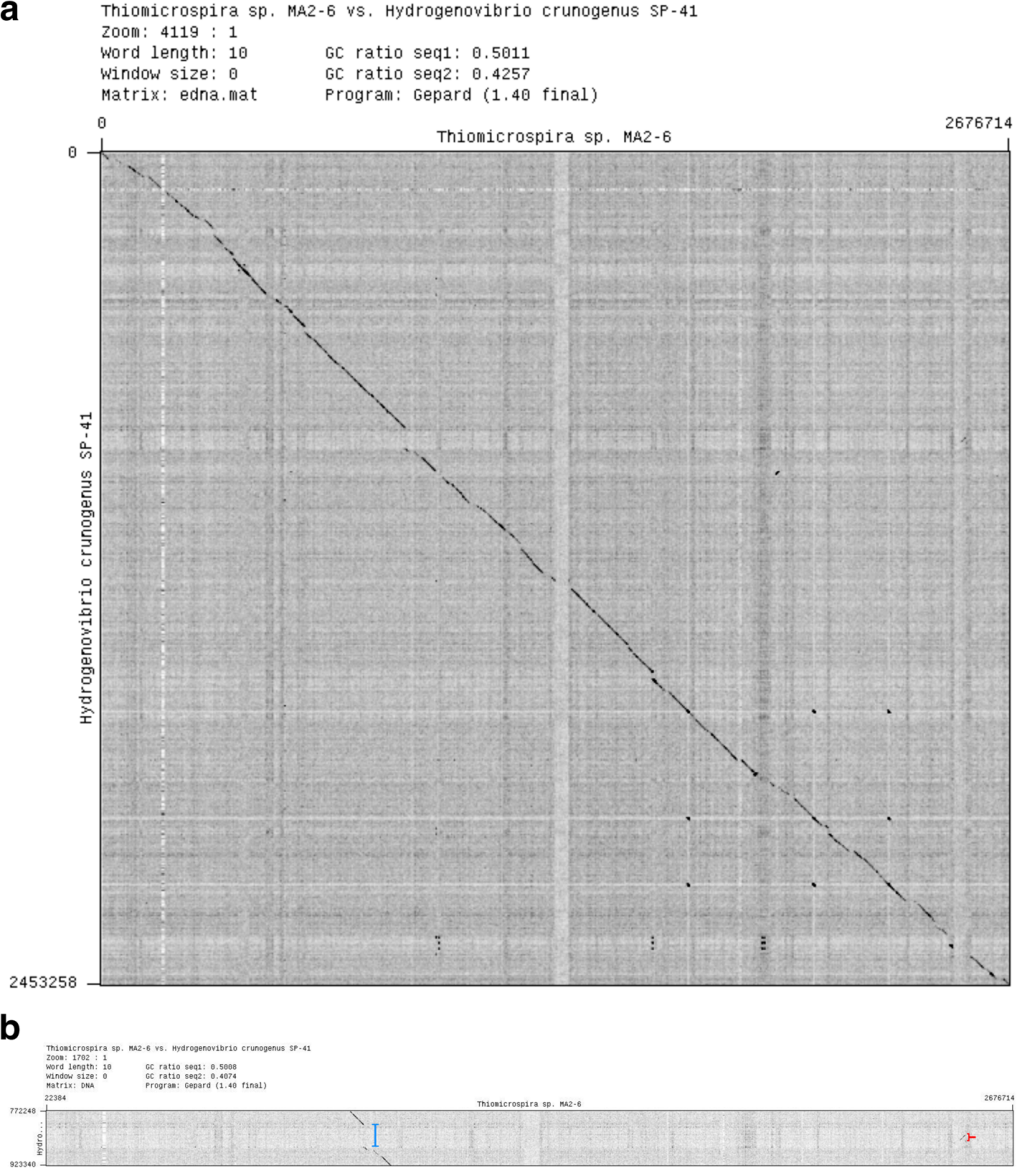
Alignment of the SP-41 and MA2-6 genomes. Dot plot of the alignment of the SP-41 and MA-2 genomes, for the whole genome **a** and detail around the hydrogenase gene cluster **b**. The orientation and starting point of the MA2-6 sequence was changed to match that of SP-41

**Fig. 6:**
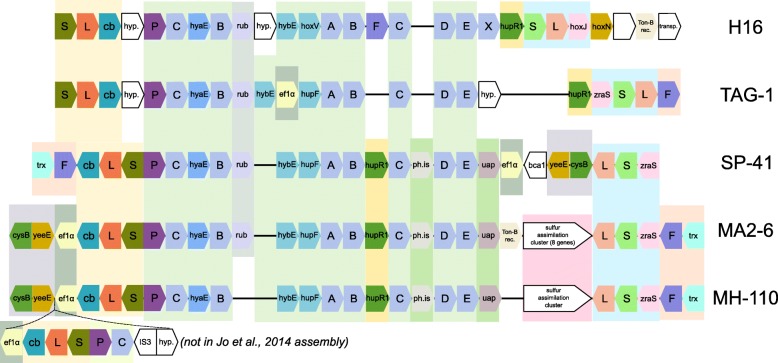
Gene order in Hydrogenase gene cluster II of SP-41 compared to homologous regions in other Proteobacteria. Comparison of the gene order in the hydrogenase gene cluster II of SP-41 with homologous regions in *Hydrogenovibrio thermophilus* MA2-6, *Hydrogenovibrio marinus* MH-110 ^*T*^, *Cupriavidus necator* H16 and *Ghiorsea bivora* TAG-1 ^*T*^. Color background rectangles are used to highlight gene syntheny. For MA2-6 and TAG-1 the reverse-complementary strand to the reference sequence is visualized, in order to maximize the number of homologs aligned to the other genomes. For MH-110, the region is different in the two available genome sequences: a gene duplication is present in the assembly by Scott et al. [21] but not in that by Jo et al. [17]

The genomic region containing the hydrogenase gene cluster II is not present in the XCL-2 genome: accordingly, only 3 of the 27 genes in the central part of the region have homologs in XCL-2 (thioredoxin; elongation factor-1-alpha; YeeE/YedE family protein; average blast hit coverage: 95.6%, similarity: 64.9%). However, several of the proteins have homologs in other organisms (Fig. 4; Additional file 12). The highest number of homologs in the region is found in the genome of *Hydrogenovibrio thermophilus* MA2-6, which has homologs of all 27 proteins in the region (average blast hit coverage: 97.1%, similarity: 80.0%). With the exception of the carbonic anhydrase, which has a homolog (coverage: 99.5%, similarity: 90.6%) elsewhere in the MA2-6 genome (cds372), most of the genes are in two regions of the MA2-6 genome (cds1576 to cds1580, cds1590 to cds1610). The genome alignment of MA2-6 to SP-41 shows that the gene order in the two genomes is mostly conserved (Fig. 5). However, the hydrogenase gene cluster is inserted in the MA2-6 genome at a different position and inverted and a region around the cluster in SP-41 is missing in MA2-6 (Fig. 5). The gene order in the hydrogenase region itself is also conserved, although some rearrangements are apparent (Fig. 6). The two groups of hydrogenase-related genes in MA2-6 are separated by 9 genes, mostly related to sulfur assimilation (sulfate adenylyltransferase; phosphoadenosine phosphosulfate reductase; sulfite reductase; cysteine desulfuration protein SufE; siroheme synthase). Of these, only SufE (encoded by MA2-6 cds1588) has a SP-41 homolog (cov: 91.8%; sim: 81.5%), encoded by a gene located elsewhere on the genome (SP-41 gene GHNINEIG_01718). The presence of genes for enzymes related to assimilatory sulfate reduction next to the hydrogenases has been postulated to be possibly assisting the synthesis of the hydrogenases iron sulfur clusters [21]. However, their absence in SP-41 shows that they are not essential for the hydrogenase activity.

Another member of the same genus, *Hydrogenovibrio marinus* MH-110, contains homologs of 25 of the 27 proteins in the region (average blast hit coverage: 97.6%, similarity: 78.5%), missing the hybE/rubredoxin and the carbonic anhydrase (which is not present in the region also in MA2-6). Two genome sequencings were performed independently by two groups ([21] and [17]). In both sequences the order of the genes in the region is very similar to that of MA2-6. The sulfur assimilation genes are also present, but not the Ton-B receptor. The MH-110 genome sequence described by [21], contains some additional genes, including a transposase and a duplication of the first hydrogenase and some of the related genes, homologous to SP-41 genes GHNINEIG_00796 to GHNINEIG_00800. However, these genes are not present in the sequence described by [17]. Besides this, the adenylyltransferase small subunit gene is not annotated by [17]. As the two sequencing projects target the same strain, it is unclear if the differences in the sequences represent a genuine rearrangement or if they are sequencing or assembling artifacts.

As XCL-2 is more closely related to SP-41, than the other two strains (Additional file 3), different reconstructions of the evolutionary history of the region remain possible. It might have been acquired by an ancestor of these bacteria and then lost by XCL-2 and other strains of *H. crunogenus*; in this case, it remains unclear for which reason the region was not maintained, as it confers a larger metabolic flexibility. Alternatively, the region might have been acquired horizontally multiple times; this was considered the most likely explanation to explain the presence of the region in strains of *H. thermophilus* and *H. marinus* [21], but not in related strains, and could also hold for SP-41. This would explain, why the island is present in different genomic surroundings. It is not known, why this particular hydrogenase island appears so well-suited for members of *Hydrogenvibrio*. A possible reason could be the presence of the group 2b sensory hydrogenase, which could confer an advantage in the regulation of hydrogenase activity in response to rapid changes in H_2_ availability.

Outside of the *Thiomicrospira* / *Thiomicrorhabdus* / *Hydrogenovibrio*, 9 other bacterial genomes contain 20 or more homologs of the region, mostly Gammaproteobacteria. The organisms with the next highest number of homologs (Fig. 4) are Gammaproteobacteria living as symbionts, i.e. the Chromatiaceae strain 2141T.STBD.0c.01a (23 homologs), symbiont of the giant shipworm *Kuphus polythalamia* [40] and Candidatus *Endolucinida thiodiazotropha* (21 homologs), symbiont of the shallow water bivalve *Codakia orbicularis* [41]. The highest number of homologs outside of the Gammaproteobacteria is found in *Thiomonas* sp. FB-Cd (Betaproteobacteria; 21 homologs). Only a few proteins have homologs in organisms outside of Proteobacteria (Additional file 12), with the highest value (7 homologs) found in the cyanobacterium *Nostoc punctiforme*.

Several genomes of known hydrogen oxidizers from hydrothermal vents have been sequenced. Among the Proteobacteria, outside of the *Thiomicrospira* / *Thiomicrorhabdus* / *Hydrogenovibrio* clade, these include the complete genomes of *Nitratifractor salsuginis* E9I37-1 ^*T*^ [42] (Iheya field, Mid-Okinawa Trough), and the draft genomes of *Caminibacter mediatlanticus* TB-2 ^*T*^ (Mid-Atlantic Ridge) [43], *Ghiorsea bivora* TAG-1 ^*T*^ (TAG site, Mid-Atlantic Ridge) and SV-108 (Snail Vents, Mariana back-arc) [44], *Nitratiruptor tergarcus* MI55-1 ^*T*^ (Iheya field, Mid-Okinawa Trough) [45] and *Hydrogenimonas thermophila* EP1-55-1 ^*T*^ (Karei field, Central Indian Ridge) [46]. Among these genomes, homologs of the SP-41 proteins of the region were found only in *Ghiorsea bivora* TAG-1 ^*T*^. The hydrogenase gene cluster of TAG-1 is almost identical to that of the other strain of the species, SV-108 [44] and is surrounded by an integrase and a recombinase. Only a few differences were found in the gene arrangement, compared to SP-41 (Fig. 6), suggesting a common origin. Among known hydrogen-oxidizing Proteobacteria isolated from other habitats, an homologous region was found to the megaplasmid pHG1 of *Cupriavidus necator* H16, which codes for four different hydrogenases [47]. The homologous region of pHG1 has a gene arrangement similar to that of *Ghiorsea bivora* TAG-1 ^*T*^ (Fig. 6). Also, in both strains, differently from SP-41 and the other *Hydrogenovibrio* strains, all genes in the region have the same orientation. Also for the H16 strain, signs of possible DNA integration are present: a transposase gene is found in close proximity (Fig. 6).

### Comparison of the functional potential of SP-41 and XCL-2

Besides the regions discussed in the previous sections (i.e. hydrogenase gene cluster II, CRISPR array, prophage) the genomes of SP-41 and XCL-2 contain several other exclusive and divergent regions. In order to assess their potential role in conferring additional metabolism abilities and other environment adaptations, we compared the COG and KO annotations of the two genomes. COG annotations (Additional file 13) and KO annotations (Additional file 14) were assigned to an amount of XCL-2 proteins (62.7% and 63.9%, respectively) very similar to that of SP-41 (64.0%; 64.2%). We identified regions coding for proteins with COG and/or KO annotations not present in the genome of the other strain. In total (without considering transposases), SP-41 contains 17 such regions, with 47 exclusive KO and 59 exclusive COG annotations (Additional file 15). Conversely, the SP-41 genome lacks 30 KO and 22 COG annotations, present in 14 exclusive or divergent regions of the XCL-2 genome (Additional file 16). Next, we describe these regions, generally following their order in the genome.

No differences to XCL-2 were observed in the citric acid cycle enzyme: i.e. as XCL-2 [14], SP-41 is also lacking 2-oxoglutarate dehydrogenase and malate dehydrogenase. SP-41 carries, similar to MA2-6 and other members of the genus, but not XCL-2 [21] enzymes for the phosphate acetyltransferase-acetate kinase pathway. These are encoded by a small insertion (genes GHNINEIG_00105 and GHNINEIG_00106) to the XCL-2 genome, together with a gene for a putative mobility protein (also present in MA2-6).

The number of membrane transporters is low in XCL-2, reflecting its obligate autotrophic lifestyle [14]. SP-41 has a similar number of KEGG orthology protein annotations included in the KEGG Brite hierarchy “Transporters” (ko02000) (172 in SP-41 and 171 in XCL-2). However, the SP-41 transport proteome covers a wider range of functions (133 KO groups vs. 123 for XCL-2). The transport systems exclusive of SP-41, described below with further detail, are those for urea (UrtABCDE), iron (AfuABC), and mercury (MerRTP), and are located in regions of the genome, which have likely been horizontally acquired.

For the uptake of nitrogen, SP-41 has, in common with XCL-2, nitrate transporter and assimilation proteins NasFED and NasA, the nitrite reductase NirBD, as well as 3 of the 4 Amt ammonia transporters of XCL-2. However, SP-41 contains also an additional region of the genome, including a gene cluster *ureDABCEFGH* for urease and its accessory proteins, genes for amidase (EC 3.5.1.4) and formamidase (EC 3.5.1.49), nitric oxide reductase activation protein NorD, and a urea transport system (genes *urtABCDE*). Some members of the genus *Hydrogenovibrio* are able to use urea as nitrogen source, e.g. *H. marinus* [48]. Urease and urea transport genes are also present in other genomes: The closest known relative to the SP-41 region is found in the *Hydrogenovibrio kuenenii* genome, which contains the *urt*, amidase and *ure* genes in the same order (although lacking the *NorD* and formamidase genes). Genes for urease are also found in the genome of *Hydrogenovibrio* sp. Milos-T1.

Despite the presence of three NorD genes in SP-41, no other components of a nitric oxide reductase operon were found. Instead, in another single-gene spanning exclusive region of the genome, SP-41 codes for a nitric oxide dioxygenase (EC 1.14.12.17) with a potential role in nitric oxide detoxification [49]. Nitric oxide dioxygenase genes have been previously shown to be particularly prone to horizontal gene transfer [50].

Recently, an iron-oxidizing strain of *Hydrogenovibrio*, SC-1 has been isolated [51]. It is unknown, if other related strains exist, which share this ability. [21] reported that none of the genomes of *Hydrogenovibrio* and related genera analyzed in their study contained genes associated with iron oxidation or reduction (*cyc2*, *mtoA*, *ompB*, *omcB*). This also holds for SP-41. However, SP-41 and XCL-2 differ in their iron transport systems. Both genomes code for the ferrous iron transporters FeoAB, and the ABC transport system TroABCD capable of transporting Zn ^2+^ and Mn ^2+^, but also Fe ^2+^ and potentially Fe ^3+^ [52]. XCL-2 contains a gene for the high affinity iron transporter EfeU [53], while SP-41 codes for the iron (III) transport proteins AfuABC. The SP-41 *afuABC* genes (genes GHNINEIG_01422 to GHNINEIG_01424) are located in a short, divergent region followed by two tRNA genes, which are common recombination and insertion points. In the corresponding position of the genome, XCL-2 contains unrelated genes, including a sarcosine oxidase (EC 1.5.3.1) operon. The *efeU* gene in XCL-2 (cds2153) is instead located in a predicted genomic island, a region of the genome, which contains multiple non-homologous sequences in the two genomes and a translocation.

A further exclusive region of SP-41 contains the *merRTPA* operon (genes GHNINEIG_02228 to GHNINEIG_02231), coding for a mercury transport system and the mercury reductase MerA. It is surrounded by transposase genes, indicating a likely horizontal acquisition. MerA has been previously described as a mercury adaptation system in other deep-sea hydrothermal vents organisms [54]. Functional *mer* operons have been characterized in several members of the Actinobacteria, Firmicutes, Beta- and Gammaproteobacteria and in *Thermus thermophilus* [55].

A thiosulfate dehydrogenase (KO K19713, EC 1.8.2.2) was annotated by KEGG BlastKoala in a genomic island region of the XCL-2 genome not present in SP-41 (cds2156, annotated in the reference sequence as “cytochrome c”). Like XCL-2, SP-41 carries two genes encoding sulfide:quinone oxidoreductase enzymes (*sqrA* and *sqrF*) reflecting its ability to consume hydrogen sulfide at different sulfide levels. Homologs to all Sox genes of XCL-2 were found in SP-41. Like in XCL-2, their arrangement differs from that typical of facultatively autotrophic sulfur-oxidizers, as the system is encoded by three groups of genes (*soxXYZA*, *soxB* and *soxCD*), located in different regions of the genome [14], which may be indicative of a differential regulation of these components [21].

A feature missing in SP-41 is the system for tRNA seleno-modification. This consists in the two genes *selD* (seledine, water dikinase, EC 2.7.9.3) and *selU*/*ybbB* (tRNA 2-selenouridine synthase), which are located in a 2-gene exclusive region of XCL-2 (cds 1052-1053). Seleno-modification occurs at tRNAs for Glu, Gln and Lys. The function of this modification is not completely understood, although it is thought to be related to the codon-anticodon interaction [56, 57].

Both genomes contain a region of the genome coding for sugar/nucleotide metabolism enzymes related to cell wall, membrane and flagellum. The region is partly non-homologous in the two genomes, although functionally related: Products of the genes in the region include 8 exclusive KO annotations for SP-41 (FlaA1, WbbJ, RmlA1, RfbB, RfbX, GlpA, OafA, MviM) and 8 exclusive KO in XCL-2 (WcaJ, ManC, Tld, Gmd, RfbC, AscC, RfbG and FbF).

## Conclusions

As previously assumed from the comparison of the 16S rRNA genes (identity ≥99%; [8]), the genome of *Hydrogenovibrio* sp. SP-41 is closely related to that of *Hydrogenovibrio crunogenus* XCL-2. Despite a low average nucleotide identity (87.7%), which would suggest an assignment of the two strains to different species, the alignment of their genomes shows a highly conserved gene order. However, additional sequences are present in both genomes, in short non-homologous regions, or insertions to one of the two genomes in traits where the rest of the sequence is collinear.

Two hydrogenase gene clusters were found in SP-41. Cluster I is homologous to the hydrogenase gene cluster in XCL-2 and codes for a group 1b hydrogenase. Cluster II is absent in XCL-2 and codes for two hydrogenases: a group 1d periplasmic membrane-anchored hydrogenase and a group 2b sensory hydrogenase. Their genomic proximity might indicate interplay of these two hydrogenases, such as regulation of the group 1d hydrogenase by the sensory hydrogenase in response to different hydrogen concentrations in the environment.

Hydrogenase gene cluster II has been likely derived from horizontal gene transfer, as it is surrounded by DNA modification and mobilization genes, and is predicted as genomic island. The closest relatives of this region are found in members of the same genus (*H. thermophilus* MA2-6, *H. marinus* MH-110 ^*T*^). As previously observed [21], it is likely that the region has been acquired multiple times in the lineage, as it is located in different genomic contexts in the different strains. If this is the case, all these strains acquired the hydrogenase from a similar source. However, horizontal acquisition of this gene cluster might be common, well beyond this lineage. Similar regions were found in hydrogen oxidizers, phylogenetically distant from SP-41 and isolated from very different and geographically distant habitats: the Betaproteobacterium *Cupriavidus necator* H16 (isolated in Germany from soil samples [58]) and two strains of the Zetaproteobacterium *Ghiorsea bivora*. The latter were isolated from similar habitats (iron mats of hydrothermal vents) but far away from each other (TAG-1 ^*T*^: TAG vent site, Mid-Atlantic Ridge; SV-108: Snail Vents site, Mariana back-arc; [44]). Also in these genomes, genes related to DNA mobility were found in proximity of their hydrogenase gene clusters.

Both MH-110 and MA2-6 are able to grow on hydrogen, as SP-41, thus likely the presence of this region explains the difference in this ability from XCL-2, which lacks the region. We showed that both the large subunit genes of cluster II group 1d hydrogenase and of cluster I group 1b hydrogenase are expressed during H_2_ consumption, and their expression is higher when H_2_ is the only available electron donor. As both hydrogenases are expressed, we hypothesize that elements of both hydrogenase gene clusters could interact in SP-41 during the observed hydrogen oxidation activity. This could explain the differences in activity rate and hydrogen affinity of SP-41 to MA2-6, both containing cluster II. However, if this is the case, this is probably not the only activity mechanism of cluster I, as interaction cannot explain its presence and conservation in XCL-2, where the cluster II is absent.

Besides the ability of growing on hydrogen, horizontal transfer of genetic material appears to play an important role in shaping the genome of SP-41. This is reflected by the higher number of transposases (compared to XCL-2) located in multiple small regions not present in the XCL-2 genome. These regions often contain signs of possible DNA mobilization, such as the presence of genes for transposases, integrases and DNA modification, or a genomic position next to common insertion points, such as tRNA genes. In an environment where DNA mobility is likely very high, the necessity may also arise to protect against unwanted sequences, such as invading plasmids or viruses; this might explain the presence of a CRISPR locus, not present in XCL-2. In a group of autotrophic Protobacteria genomes the average number of transposases appears to be higher where CRISPR loci are present. However, we found also several counter-examples, where presence of CRISPRs and abundance of transposases are not correlated. Thus, further studies are necessary to understand the observed correlation and which other factors may play a role.

The inserted DNA confers to SP-41 features absent in XCL-2, such as a urease and transport system for urea, a transport system for ferrous iron and a detoxification system for mercury. These may be important for the survival in the specific environment. Adaptations to the habitat are a common feature of *Thiomicrospira*, *Hydrogenovibrio* and *Thiomicrorhabdus* species, explaining their prevalence in multiple heterogeneous environments [21]. Besides this, as postulated for other hydrothermally influenced habitats, high levels of horizontal gene transfer may confer an advantage to the bacterial community as a whole [28].

## Methods

### Cultivation of SP-41 isolate and enrichment

An enrichment culture containing SP-41 as well as the isolated strain SP-41 were previously obtained and cultivated in our laboratory [9]. For later DNA isolation, the *Hydrogenovibrio* sp. SP-41 isolate was grown in 400 ml T-ASW with a pH of 7.5-7.8 [59] in 1l flasks at 28 ^∘^C for approximately 2.5 days. The pH decrease of the medium, inoculated with a fresh pre-culture, was monitored by the color change of the phenol red contained in the medium. When necessary, the pH was increased with a 5% NaHCO_3_ solution. The cells were harvested by centrifugation at 17,000 g (Sorvall TC 6 Plus Centrifuge, Thermo Fisher Scientific Inc.), washed in 1x PBS buffer and pelleted again by centrifugation. The cell pellet was stored at -20 ^∘^C until further use. An additional purity check of the culture was performed with fluorescence in situ hybridization (FISH) as described before [9], showing a SP-41 specific probe signal for every DAPI-stained cell.

Additionally, the enrichment culture dominated by *Hydrogenovibrio* sp. SP-41 was grown in 120 ml serum bottles filled with 50 ml of MJ medium. The headspace of the bottles was replaced by a gas mixture of H_2_:CO_2_:O_2_ (79:20:1; Westfalen AG, Münster, Germany) as stated before [9]. The culture was incubated for approximately five weeks at 28 ^∘^C under weekly regassing of the head space. After harvesting three liters of the culture at 17,000 g (Sorvall TC 6 Plus Centrifuge, Thermo Fisher Scientific Inc., Waltham, MA, USA), sedimented cells were washed in 50 mM Tris buffer (pH 8.0). The repelleted cells were stored at -20 ^∘^C until further use.

### Analysis of the expression of the [NiFi]-hydrogenase genes

For the qRT-PCR experiments, *Hydrogenovibrio* SP-41 was grown in MJ (1400 mL per biological replicate) and MJ-T media (700 mL per biological replicate) with an atmosphere of H_2_:CO_2_:O_2_:He(2:20:1:78*%*(*v*/*v*)) as described in [9]. Cells were harvested after 8 and 24 h of incubation at 28 ^∘^C by centrifugation at 17,000 g and 4 ^∘^C for 30 min (Sorvall LYNX 4000, Thermo Scientific, Waltham, MA, USA). After a washing step with 1.5 mL 1x PBS buffer, cell pellets were stored at -80 ^∘^C. For isolation of total RNA, the cell pellets were resuspended in 500 *μ*L TriReagent (Zymo Research, Irvine, CA, USA), transferred to ZR Bashing Bead Lysis Tubes (0.1-0.5 mm, Zymo Research) and cells were lysed by vortexing at full speed for 10 min. RNA was purified from the cell lysates using the Direct-zol™RNA Miniprep Kit (Zymo Research) according to the manufacturer’s instructions, followed by an additional DNase treatment with the DNase Max Kit (Qiagen, Hilden Germany). The total (DNA-free) RNA was transcribed into cDNA using the SuperScript™VILO™Master Mix (Invitrogen, Carlsbad, CA, USA) according to the manufacturer’s instructions with up to 2.5 *μ*g RNA. The resulting cDNA was purified with the DNA clean and concentrator-5 Kit (Zymo Research) and eluted with 20 *μ*L of elution buffer (10 mM tris-HCl, pH 8.5). The following primer pairs were used to perform qRT-PCR experiments, yielding products of ≈ 150 bp: (i) *rpoD-1337F*(5’-ACCGTATTCAGCGTCAGTTG-3’) and *rpoD-1476R* (5’-TGGCGTTTCCATTGAGATCG-3’) to amplify the housekeeping gene *rpoD*, (ii) *40F* and *189R* (see [9]) for the amplification of the gene for the large subunit of the group 1b hydrogenase in hydrogenase gene cluster I and (iii) *hynL2-1260F* (5’-CGCACAAGGTGTTGAGTACG-3’) and *hynL2-1409R* (5’-GCTCGGGCTAAAGTTCTTCC-3’) to amplify the gene for the large subunit of the group 1d hydrogenase in hydrogenase gene cluster II. The amplification was performed using the SYBR™Select Master Mix (Applied Biosystems, Foster City, CA, USA) with 25 ng of cDNA as template in a 20 *μ* L reaction. The qPCR was run in a C1000 Touch Thermal Cycler equipped with a CFX 96 Real Time System (Bio-Rad Laboratories Inc., Hercules, CA, USA) under the following conditions: initial denaturation at 98 ^∘^C for 2 min; 40 cycles of 98 ^∘^C for 15 s, 52 ^∘^C for 20 s and 72 ^∘^C for 30 s. Non-template as well as non-RT (i.e. RNA) controls were performed for each primer pair in every run. Three technical and biological replicates, each, were performed for the MJ-T samples. For the MJ media samples, only the 24 h incubation of one sample yielded sufficient RNA and cDNA material. Therefore, only one biological replicate (with three technical replicates) could be analyzed for this condition. The inter-run comparability was ensured by repeating a reaction (in triplicate) on the next plate as calibrator. The relative quantities of expressed hydrogenase genes were calculated and normalized to the single-copy housekeeping gene *rpoD*. Technical and biological replicates were arithmetically averaged and an overall mean value was calculated. Standard deviations of the technical replicates were propagated forward by applying the Gaussian propagation of error to calculate the error of the overall mean values.

### DNA extraction and sequencing of SP-41 enrichment and isolate

The DNA isolation of SP-41 was performed using the MagAttract HMW DNA Kit (Qiagen, Hilden, Germany) according to the manufacturer’s instructions. Residual RNA was removed via digestion with 10 *μ*/ml of DNase-free Rnase A (Applichem GmbH, Darmstadt, Germany) for 2 h at room temperature. The DNA was subsequently purified using the SureClean Plus Kit (Bioline GmbH, Luckenwalde, Germany) according to the manufacturer’s protocol but avoiding the co-precipitant. The purity of the DNA was checked by PCR amplification of the 16S rRNA gene as well as *hynL* genes, followed by cloning and sequencing of the PCR products as described before [9]. The DNA of the SP-41 isolate was sequenced using the PacBio RSII technique (Pacific BioSciences, Menlo Park, CA, USA) at GATC Biotech AG (Konstanz, Germany). DNA of the enrichment culture was isolated according to Böhnke and Perner [60] and the presence of SP-41’s DNA was checked analogously to the purity check of the DNA of the SP-41 isolate. Two DNA libraries (a mate-pair and a fragment library) were constructed and paired-end sequenced by Microsynth AG (Balgach, Switzerland) using the Illumina MiSeq platform (Illumina, San Diego, CA, USA). Quality control for the sequencing reads of both approaches was performed using FastQC v. 0.11.5 [61].

### Assembly of the SP-41 genome

The SP-41 Pacific Biosciences reads were assembled using Canu v. 1.6 [62] using an estimated genome size parameter of 2.8 Mbp. Using circlator v. 1.5.2 [63] with default parameters, the assembly was circularized and oriented to start from the *dnaA* gene. The Illumina reads obtained from the SP-41 enrichment culture were mapped to the assembly using bwa mem v. 0.7.15 [64], with default parameters. The resulting alignments were sorted and indexed using samtools v. 1.4.1 [65]. Using Pilon v. 1.22 [66] with the parameters –fix all and –mindepth 0.5, the alignments were used to correct the assembly. This was performed in three subsequent steps, using paired end reads from the fragment library in the first step, reads classified as paired end from the mate pair library in the second step, and reads classified as mate pair reads from the mate pair library in the third step.

### Annotation of the SP-41 genome

The genomic sequence of SP-41 was first annotated using the Prokka pipeline v. 1.12 [22] using the option –compliant. The protein sequences from all annotated genes for which the product annotation was “hypothetical protein” were aligned to the RefSeq Protein database by Blast. Only results with query coverage of at least 80% and a maximum e-value of 10^−5^ were considered. Hits to proteins whose product description was “hypothetical protein” or contained one of the strings “predicted protein”, “unknown function” or “domain-containing protein” were filtered out. For each of the queries, all remaining hits to proteins of XCL-2, MA2-6 and the highest score hit among all others were retained. The matching proteins in this hit set were extracted from the Refseq Protein database and used as a custom protein database for a second pass of annotation using Prokka. In the final Prokka annotation, 10 product descriptions of CDS features contained the word “partial”, not allowed by Genbank; the word was removed and the product was described as putative.

### Alignment to Refseq Protein

The Blast database of all NCBI Refseq proteins was obtained on 2017/10/19 using update_blastdb.pl from NCBI Blast+ v.2.7.0 suite [67]. The protein sequences were aligned to the database using blastp v. 2.7.0 with default parameters. Hits with query coverage smaller than 80% or an e-value higher than 10^−5^ were discarded.

### Comparison to related genomes

The sequence (Fasta) and annotation (GFF3) of other related bacterial genomes were obtained from the NCBI Refseq database [68], with the following accession numbers: *Hydrogenovibrio crunogenus* XCL-2: NC_007520.2; *Hydrogenovibrio thermophilus* MA2-6: NZ_JOMK01000001.1; *Hydrogenovibrio marinus* MH-110 ^*T*^/DSM11271 ^*T*^: NZ_JOML01000001.1 to NZ_JOML01000003.1; *Thiomicrospira aerophila* AL3 ^*T*^: NZ_CP007030.1; *Thiomicrospira cyclica* ALM1 ^*T*^: NC_015581.1; *Hydrogenovibrio halophilus* DSM 15072 ^*T*^: NZ_KB913033.1; *Hydrogenovibrio* sp. Milos-T1: NZ_JQMT01000001.1; *Thiomicrospira pelophila* DSM 1534 ^*T*^: NZ_JOMR01000001.1. The pairwise average nucleotide identity (ANI) of these genomes was computed by Jspecies v. 1.2.1 [69] using the ANIb algorithm [25].

### Comparison of the arrangement of hydrogenase gene cluster II

We compared the arrangement of the genes in SP-41 hydrogenase gene cluster II with that of the similar clusters in MA2-6 and MH-110 (organisms with the highest number of homologs in the region). Furthermore we also included in the comparison other related genomic sequences: a second assembly of the *Hydrogenovibrio marinus* MH-110 ^*T*^ genome, described by [17] (GenBank, accession JMIU01000000), the megaplasmid pHG1 of *Cupriavidus necator* H16 (Refseq NC_005241.1) and the genomes of *Ghiorsea bivora* TAG-1 ^*T*^ (Refseq NZ_JQLW00000000.1), *Nitratifractor salsuginis* E9I37-1 ^*T*^ (Refseq NC_014935.1) *Caminibacter mediatlanticus* TB-2 ^*T*^ (Refseq NZ_ABCJ00000000.1), *Nitratiruptor tergarcus* MI55-1 ^*T*^ (Refseq NZ_FWWZ00000000.1) and *Hydrogenimonas thermophila* EP1-55-1 ^*T*^ (Refseq NZ_FOXB00000000.1). Among these, only genomes where an inspection of the annotations in proximity of the hydrogenase clusters revealed a similar structure to that of hydrogenase gene cluster II of SP-41 were further considered (strains TAG-1 and H16). For the illustration, we manually re-annotated the gene names and functions on the base of the BLASTp alignments of their products to the SP-41 and NCBI Refseq proteins.

### Annotation of genomic islands

Lists of genomic islands predictions of were obtained from of IslandViewer4 [26], from the database of pre-computed results (for XCL-2, accession NC_007520.2) or computed using the interactive web application (for the SP-41 genome). Thereby the results for XCL-2 are based on merging the pre-computed predictions by IslandPath-DIMOB [27] and Islander [70], while for uploaded genomes only IslandPath-DIMOB predictions are computed. Therefore we run the standalone version of Islander v. 1.2 with default parameters on the SP-41 genome; as it did not predict any further island, no merging was necessary.

### Pairwise alignment of genome sequence and annotation

Dot plots of the alignments of the SP-41 genome against other related genomes were obtained using Gerard v. 1.4 [71] using default parameters. Pairwise alignments of the SP-41 genome against other related genomes were computed using progressiveMauve [72] using default parameters. Using an custom python script, each region of the Mauve alignment of SP-41 and XCL-2 was classified in common or exclusive of one of the two genomes. For common regions, collinear features were identified based on their position and on BLAST alignments of the protein sequences of the two genomes. Thereby only hits with query coverage of 80% and maximal e-value of 10^−5^ were considered.

### Comparative functional annotation

The assignment of KO annotations to the proteins of the SP-41 and the XCL-2 genomes was performed using KEGG Blastkoala [24], selecting the prokaryotes taxonomy group and the species_prokaryotes database. Differences in KO annotations of orthologs in the two strains where eliminated by transferring the KO annotation among the homologs found by blastp alignment. The results were mapped to the KEGG Pathways, Brite terms and Modules ontologies using KEGG mapper v. 3.1 [73]. The assignment of COG annotations to the proteins of the SP-41 and the XCL-2 genomes was performed using CD-search [23], selecting the COG database [74] and using default parameters. Sets of common and exclusive annotations were identified using custom Python scripts.

### Circular plot of the SP-41 genome

A circular plot of the SP-41 genome was created using Circos v. 0.64 [75]. The data for the GC% plot track was computed by a custom Ruby script as average value in windows of 128 nucleotides along the genome. Functional categories for the protein-coding genes were computed from the CD-search COG assignments, using the mapping (cognames2003-2014.tab) and the names of the functional categories (fun2003-2014.tab) available in the NCBI FTP server at https://ftp://ftp.ncbi.nih.gov/pub/COG/COG2014/data/.

### Number of tranposases and CRISPRs presence in autotrophic Proteobacteria

A list of autotrophic Protobacteria genomes and a list of transposase PFAM [76] families (pfam00872, pfam01527, pfam01609, pfam01797, pfam02371, pfam05598, pfam09299, pfam12762, pfam12784) were obtained from [21]. The presence or absence of a CRISPR annotation and the sum of the number of transposases in the PFAM families were obtained from IMG/M [77]. The hypothesis that the number of transposases for genomes with a CRISPR was from a distribution with a higher mean than that for genomes without a CRISPR was tested using a Welch’s one-sided t-test as implemented by the R function t.test, with the parameters var.equal=N,alternative="greater". The significance level was set to 0.05.

### Multiple sequence alignment of the 16S rRNA genes

The three 16S rRNA genes of SP-41 (GHNINEIG_01594, GHNINEIG_01882, GHNINEIG_02064), XCL-2 (rna38, rna45, rna53) and *Escherichia coli* K-12 (obtained from EcoCyc [78], accession EG30084) were aligned using muscle v. 3.8.31 [79]. As the three copies of XCL-2 were identical, only one was retained in the final alignment. The position of hypervariable regions was annotated in the alignment based on the coordinates of the regions in the *E. coli* K-12 sequence [80].

## Additional files

Additional file 1 COG annotations of SP-41 proteins. COG assignments by CD-search [23] using the COG database [74] to the proteins encoded by the SP-41 genome. COG annotations were assigned to 64.0% of the SP-41 proteins. (CSV 44 kb)

Additional file 2 KO annotations of SP-41 proteins. KO assignments by Blastkoala [24], using the prokaryotes taxonomy group and the species_prokaryotes database to the proteins encoded by the SP-41 genome. KO annotations were assigned to 64.2% of the SP-41 proteins. (CSV 118 kb)

Additional file 3 Pairwise average nucleotide identity. Pairwise average nucleotide identity (ANI) computed using the ANIb algorithm, of the genome of SP-41 and related genomes. (CSV 1 kb)

Additional file 4 Best Blast hit in XCL-2 proteins for each protein of SP-41. Table reporting the results of the alignment of SP-41 proteins to the XCL-2 proteins using BlastP. Only the best hit (if any) for each SP-41 protein is reported. (CSV 314 kb)

Additional file 5 Alignment of the 16S rRNA genes of SP-41 and XCL-2. Multiple sequence alignment of the three 16S rRNA genes of SP-41, XCL-2 (in a single sequence, as its 3 copies are identical) and *E. coli* K12. The borders of the hypervariable regions in the alignment were annotated, based on their coordinates in the *E. coli* sequence. (TXT 17 kb)

Additional file 6 Base calling errors of previous SP-41 16S rRNA sequencing. Initial part of the chromatogram of the 16S rRNA gene sequencing of SP-41 with the 26F primer, described in [9]. The regions with a light red background contain bases which are different in one of three 16S rRNA gene copies of SP-41. The base calling was assuming that the sequence was in single copy, thus called the most common base. From 5’, this happened in 1 position in the first highlighed region, 4 positions in the second highlighed region and 2 positions in third highlighed region. (PDF 1801 kb)

Additional file 7 Collinear, divergent, exclusive and translocated regions of the alignment of the SP-41 and XCL-2 genomes. Coordinates of the regions of the alignment of the genomes of SP-41 and XCL-2, classified as collinear (same gene order), translocated (same gene order but moved in another genomic context), divergent (two different regions present in the two genomes, with different genes), exclusive (additional region with at least one annotated feature present only in one of the two genome, while in the other genome the region is absent, or a different sequence, with no annotated features is present). (CSV 16 kb)

Additional file 8 Coordinates of the genomic islands of SP-41 and XCL-2. Coordinates of the 7 genomic islands in the SP-41 genome and of the 9 genomic islands in the XCL-2 genome, as predicted by IslandViewer [26]. (CSV 1 kb)

Additional file 9 Coding sequences of SP-41 overlapping genomic islands. Coordinates and product description of coding sequence (CDS) annotations of the SP-41 genome partially or completely overlapping genomic islands predicted by IslandViewer [26]. (CSV 12 kb)

Additional file 10 Coding sequences of XCL-2 overlapping genomic islands. Coordinates and product description of coding sequence (CDS) annotations of the XCL-2 genome partially or completely overlapping genomic islands predicted by IslandViewer [26]. (CSV 19 kb)

Additional file 11 Presence of CRISPR loci and transposase abundance in genomes of a group of autotroph Proteobacteria. Presence of CRISPRs and transposases belonging to Pfam families previously selected for a similar analysis by [21] in a set of genomes of autotroph proteobacterial strains, described in the same study. The annotation data (number of CRISPRs and Pfam annotations) was obtained from the IMG/M platform [77]. The file also contains a transcript of the R session in which the statistical significance of the differences was assessed.(CSV 7 kb)

Additional file 12 Homologs of the Hydrogenase Gene Cluster II proteins. Hits by BlastP in the NCBI Refseq Protein database of the protein encoded by the Hydrogenase Gene Cluster II region of the SP-41 genome. (CSV 1389 kb)

Additional file 13 COG annotations of XCL-2 proteins. COG assignments by CD-search [23] using the COG database [74] to the proteins encoded by the XCL-2 genome. COG annotations were assigned to 62.7% of the XCL-2 proteins. (CSV 42 kb)

Additional file 14 KO annotations of XCL-2 proteins. KO assignments by Blastkoala [24], using the prokaryotes taxonomy group and the species_prokaryotes database to the proteins encoded by the XCL-2 genome. KO annotations were assigned to 63.9% of the XCL-2 proteins. (CSV 115 kb)

Additional file 15 Regions of the SP-41 genome with exclusive KO/COG annotations. Regions of the SP-41 genome containing genes coding for protein assigned to ortholog groups (KO, COG) not present in XCL-2. (PDF 86 kb)

Additional file 16 Regions of the XCL-2 genome with exclusive KO/COG annotations. Regions of the XCL-2 genome containing genes coding for protein assigned to ortholog groups (KO, COG) not present in SP-41. (PDF 83 kb)

## Abbreviations

COG: Clusters of Orthologous Groups
CRISPR: clustered regularly interspaced short palindromic repeats
KO: KEGG Orthology
qRT-PCR: quantitative reverse transcriptase polymerase chain reaction
