# Supplementary figures and images for: Horizontal acquisition of hydrogen conversion ability and other habitat adaptations in the Hydrogenovibrio strains SP-41 and XCL-2
Source: BMC Genomics. 2019 May 6;20:339. doi: 10.1186/s12864-019-5710-5 (PMC6501319; doi:10.1186/s12864-019-5710-5)

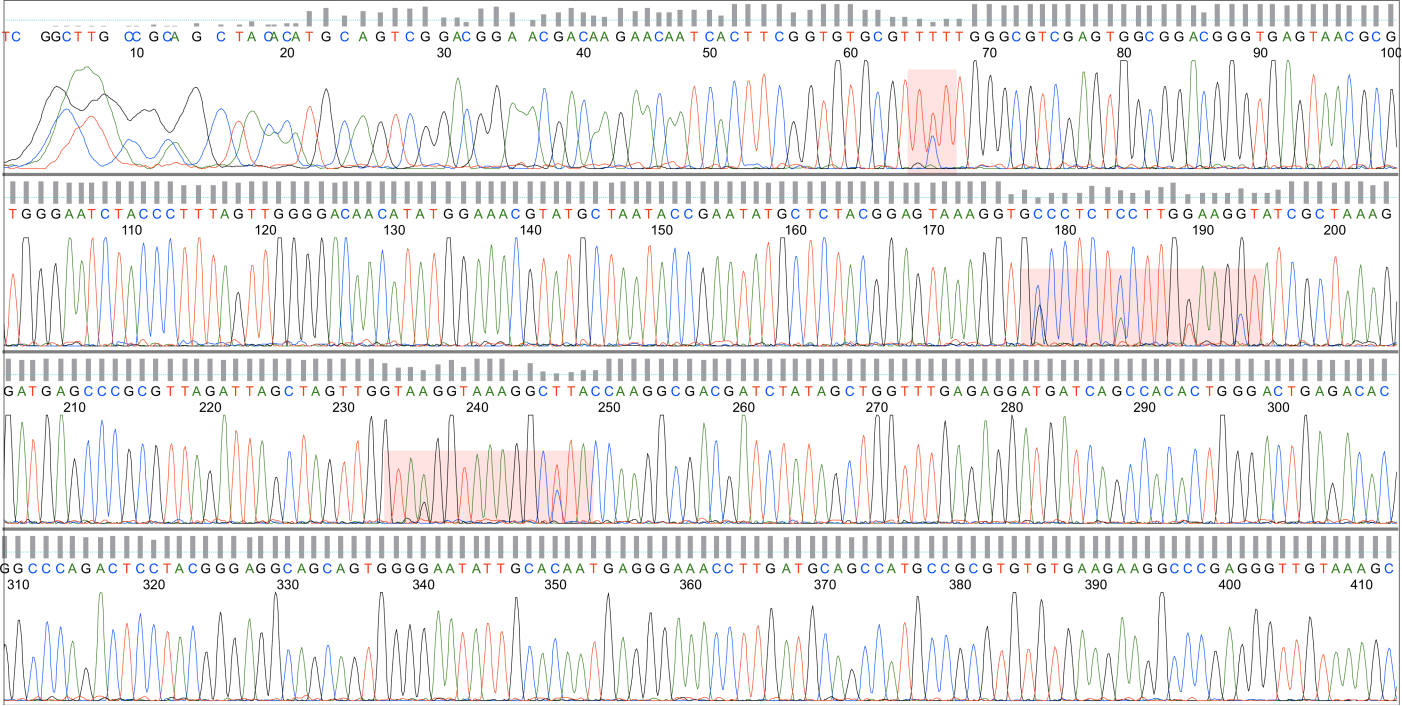

Supplement: Supplementary file 6 — Base calling errors of previous SP-41 16S rRNA sequencing. Initial part of the chromatogram of the 16S rRNA gene sequencing of SP-41 with the 26F primer, described in [9]. The regions with a light red background contain bases which are different in one of three 16S rRNA gene copies of SP-41. The base calling was assuming that the sequence was in single copy, thus called the most common base. From 5’, this happened in 1 position in the first highlighed region, 4 positions in the second highlighed region and 2 positions in third highlighed region. (PDF 1801 kb) [file 12864_2019_5710_MOESM6_ESM.pdf]
