# Supplementary material for: Horizontal acquisition of hydrogen conversion ability and other habitat adaptations in the Hydrogenovibrio strains SP-41 and XCL-2
Source: BMC Genomics. 2019 May 6;20:339. doi: 10.1186/s12864-019-5710-5 (PMC6501319; doi:10.1186/s12864-019-5710-5)
Supplement: Supplementary file 15 — Regions of the SP-41 genome with exclusive KO/COG annotations. Regions of the SP-41 genome containing genes coding for protein assigned to ortholog groups (KO, COG) not present in XCL-2. (PDF 86 kb) [file 12864_2019_5710_MOESM15_ESM.pdf]

#	Genomic region		Exclusive ortholog groups		Function / Notes
	SP-41	XCL-2	KO	COG	
1	3 additional genes (104-106)	not present	K00925<br>K13788	COG0280<br>COG0282/0857	phosphate acetyltransferase-acetate kinase pathway<br>(ackA; acetate kinase [EC:2.7.2.1]; pta; phosphate acetyltransferase [EC:2.3.1.8])
2	divergent<br>(genes 342-345)	exclusive part of island<br>(cds 331-341)	K19591	[COG0789]	cueR; MerR family transcriptional regulator, copper efflux regulator
3	genomic island<br>(genes 391-404)	not present	K00558	[COG0270]	DNMT1; DNA (cytosine-5)-methyltransferase 1 [EC:2.1.1.37]
4	CRISPR<br>(genes 421-423)	not present	K09951<br>K09952<br>K15342	COG3512*<br>COG3513*<br>COG1518	CRISPR-associated proteins (cas9/cas1/cas2)<br>followed by CRISPR with 22 repeats
5	18 additional genes (479-496)	not present	K01428-30<br>K03187-90	COG0804<br>COG0831-2	urease and accessory proteins (UreDABCEFG)
			K01426<br>K01455	 COG2421*	amidase [EC:3.5.1.4]<br>formamidase [EC:3.5.1.49]
			K11959-63	COG0410<br>COG4177<br>COG0559	transport system; different KO/COG annotation:<br>- KO: urea transport system (UrtEDCBA)<br>- COG: high affinity branched-amino acid transport system (LifFMH),
6	hydrogenases genomic island<br>(genes 767-829)	not present	 K07313	COG3177<br>[COG0639]	Fic family protein;<br>Ser/Thr protein phosphatase 1 [EC:3.1.3.16]
			K03618-20<br>K06281-82<br>K19641/61	COG0375	Two hydrogenases and related proteins (hyaCBAEF); hupR/hupT two-component system.<br>Note: another hydrogenase gene cluster is common to the two genomes (with K03605; K05922/7; K04651-6).
			K13634	[COG0583]	cysB; LysR family transcriptional regulator, cys regulon transcriptional activator
7	single additional gene (850)	not present	K05916	COG1017<br>COG1018	hmp; nitric oxide dioxygenase [EC:1.14.12.17]<br>*additional COG hit: fpr; Ferredoxin-NADP reductase [EC 1.18.1.2]*

8	divergent<br>(genes 1422-1424)	unrelated exclusive (cds 1372-1387)	K02010<br>K02011<br>K02012	COG1840<br>COG1178<br>COG3842	AfuABC iron(III) transport system
10	divergent (genes 1601-1603)	O-antigen polymerase (cds1556)	K01784	COG1087	galE; UDP-glucose 4-epimerase [EC:5.1.3.2]<br><br>Note: in both genomes region contains (partly different) genes related to amino sugar, nucleotide and liposaccharide metabolism
11	single additional gene (1634)	not present	K01740	COG2873	metY; O-acetylhomoserine (thiol)-lyase [EC:2.5.1.49]
12	divergent<br>(genes 1762-1783)	different, functionally related genes (cds 1721-1737)	K07154	COG3550*	hipA; Ser/Thr-protein kinase HipA [EC:2.7.11.1]
			 <br><br>K00973<br>K01710<br><br>K00111	 COG1086<br>COG0110<br>COG1209<br>COG1088<br>COG2244<br>COG0578<br>COG1835<br>COG0673	Protein mostly related to cell wall / membrane / flagellum:<br>- flaA1; NDP-sugar epimerase<br>- wbbJ; dTDP-3-amino-3,6-dideoxy-α-D-galactopyranose 3-N-acetyltransferase<br>- rmlA1: glucose-1-phosphate thymidylyltransferase [EC:2.7.7.24]<br>- rfbB; dTDP-glucose 4,6-dehydratase [EC:4.2.1.46]<br>- rfbX; O-antigen translocase<br>- glpA; glycerol-3-phosphate dehydrogenase [EC:1.1.5.3]<br>- oafA; Peptidoglycan/LPS O-acetylase<br>- mviM; putative 4,5-dihydroxyphtalate dehydrogenase
13	genomic island (genes 1903-1918)	not present	 K07154	COG3177<br>COG3550<br>COG1961	Fic family protein<br>hipA; Ser/Thr-protein kinase [EC:2.7.11.1]<br>PinE; Site-specific recombinase related to DNA invertase Pin
14	2 additional genes (2151-2152)	not present		COG0631	PrpC; Ser/Thr-protein kinase 1 [EC:3.1.3.16]
15	genomic island; partly common (2195-2218)	(cds 2123-2160)	K06039	COG1553	DsrE; Sulfurtransferase complex TusBCD TusD component
16	8 additional genes (2227-2234)	not present	K08363-5<br>K00520	[COG0789]<br>[COG2608]	Hg detoxification system MerRTAP
17	6 additional genes (2266-2271)	not present	 K09701<br>K00574	COG2907<br>COG3486*<br>COG2230	Predicted NAD/FAD-binding protein;<br>uncharacterized protein (DUF1365 family)<br>cfa; cyclopropane-fatty-acyl-phospholipid synthase [EC:2.1.1.79]

-	different regions		K07483	COG2963<br>COG2801<br>COG3328	transposases:<br>- IS3 family (9 genes: 344, 394, 1217, 1904, 1917, 2195, 2214, 2233, 2255)<br>- IS3 family (11 genes: 345, 393, 1218, 1903, 1918, 2196, 2198, 2213, 2227, 2234, 2254)<br>- IS256 family (8 genes: 392, 587, 768, 778, 821, 1125, 1487, 1875)
