# Supplementary material for: Horizontal acquisition of hydrogen conversion ability and other habitat adaptations in the Hydrogenovibrio strains SP-41 and XCL-2
Source: BMC Genomics. 2019 May 6;20:339. doi: 10.1186/s12864-019-5710-5 (PMC6501319; doi:10.1186/s12864-019-5710-5)
Supplement: Supplementary file 16 — Regions of the XCL-2 genome with exclusive KO/COG annotations. Regions of the XCL-2 genome containing genes coding for protein assigned to ortholog groups (KO, COG) not present in SP-41. (PDF 83 kb) [file 12864_2019_5710_MOESM16_ESM.pdf]

#	Genomic region		Exclusive ortholog groups		Function / Notes
	**XCL-2**	**SP-41**	**KO**	**COG**	
1	10 additional genes (cds 31-40)	not present	K19000<br>K02584<br>K05297	COG4568*	rof; Rho-binding antiterminator;<br>nifA; Nif-specific regulatory protein;<br>rubB; rubredoxin---NAD+ reductase [EC:1.18.1.1]
2	genomic island, partly common (cds 82-93)	(genes 73-77)		COG4688	uncharacterized protein
3	exclusive part of genomic island (cds 331-338)	divergent (genes 342-345)		COG4688<br>COG3472/1479<br>COG1680	integrase<br>uncharacterized protein (ParB-like and HNH nuclease domains)<br>AmpC; beta-lactamase class C family
4	genomic island (cds 368-383)			COG4916	uncharacterized protein
5	prophage, (cds 666-722)	not present	K06903<br>K06905-8<br>K06919	COG4643<br>COG5525<br>COG5511<br>COG3497<br>COG3500	phage proteins / uncharacterized proteins
6	divergent (cds 1043-1044)	gene 1081 (diguanylate cyclase)		COG2249	MdaB; NADPH-quinone reductase
7	2 additional genes (cds 1052-1053)	not present	 K01008<br>K06917	 COG0709<br>COG2603*	tRNA seleno-modification:<br>- selD; selenide, water dikinase [EC:2.7.9.3];<br>- selU; tRNA 2-selenouridine synthase [EC:2.9.1.-]
8	3 additional genes (cds 1102-1104)	not present	K01286	COG1680<br><br>COG1266	D-alanyl-D-alanine carboxypeptidase [EC:3.4.16.4]<br>*Note: EC not exclusive; other, common, KOs with the same EC: K03587/7259/7258/5515*<br>CPBP family intramembrane metalloprotease
9	divergent (cds1351-1355)	(genes 1399-1405)		COG2020	Ste14; protein-S-isoprenylcysteine O-methyltransferase

10	divergent<br>(cds 1372-1387)	Afu operon<br>(genes 1422-1424)	K22081-3<br><br>K00302<br>K00303<br>K00304<br>K00305	 <br>COG0404<br>[COG0665]<br>COG4311*<br>COG4583*	methylamine-glutamate N-methyltransferase [EC:2.1.1.21] (subunits A,B,C)<br><br><br>sarcosine oxidase [EC:1.5.3.1]<br>(subunits α, β, γ, δ)
11	divergent<br>(cds 1721-1737)	different, functionally related genes (1762-1783)	K03606<br><br><br>K00971<br>K22252<br>K01711<br>K01790<br>K12452<br>K01709<br>K00978	[COG2148]<br><br><br>COG0836/0662<br>[COG0451]<br>COG1089<br>COG1898<br>[COG0399]<br>[COG0451]<br>[COG1208]	wcaJ; putative colanic acid biosysnthesis UDP-glucose lipid carrier transferase<br><br>Protein mostly related to cell wall / membrane / flagellum:<br>- manC; mannose-1-phosphate guanylyltransferase [EC:2.7.7.13];<br>- tld; GDP-6-deoxy-D-talose 4-dehydrogenase [EC:1.1.1.135];<br>- gmd; GDP-mannose 4,6-dehydratase [EC:4.2.1.47];<br>- rfbC; dTDP-4-dehydrorhamnose 3,5-epimerase [EC:5.1.3.13];<br>- ascC; CDP-4-dehydro-6-deoxyglucose reductase, E1 [EC:1.17.1.1];<br>- rfbG; CDP-glucose 4,6-dehydratase [EC:4.2.1.45];<br>- fbF; glucose-1-phosphate cytidylyltransferase [EC:2.7.7.33]
12	single additional gene (cds1919)	not present	K03892	[COG0640]	arsR; ArsR family transcriptional regulator, arsenate/arsenite/antimonite-responsive transcriptional repressor
13	genomic island, partly common (cds 2142-2174)	(genes 2249-2262)	K07243<br>K19713	COG0672*<br>[COG3258]<br>COG3391	FTR; high-affinity iron transporter<br>tsdA; thiosulfate dehydrogenase [EC:1.8.2.2]<br>YncE; DNA-binding beta-propeller fold protein YncE
14	2 additional genes (cds2233-2234)	not present	[K07727]	COG3655	YozG; DNA-binding transcriptional regulator
